# Diabetes sepsis on Wistar rat strain (*Rattus norvegicus*) induced by streptozotocin and bacteria *Staphylococcus aureus*

**DOI:** 10.14202/vetworld.2019.849-854

**Published:** 2019-06-19

**Authors:** Dahliatul Qosimah, Dhita Evi Aryani, Ma. Asuncion Guiang Beltran, Aulanni’am Aulanni’am

**Affiliations:** 1 Laboratory of Microbiology and Immunology, Faculty of Veterinary Medicine, Brawijaya University, Indonesia; 2 Laboratory of Pharmacology, Faculty of Veterinary Medicine, Brawijaya University, Indonesia; 3 Department of Microbiology and Public Health, College of Veterinary Medicine, Tarlac Agricultural University, Camiling, Tarlac, Philippines; 4 Laboratory of Biochemical, Faculty of Veterinary Medicine, Brawijaya University, Indonesia

**Keywords:** animal model, diabetes, inflammation, sepsis

## Abstract

**Background and Aim:**

Sepsis is characterized by loss of control of the inflammatory response, which can be triggered by various microorganisms and toxic secretions. The mortality rate increases due to impaired endothelial function caused dysfunctional organ systems. Diabetes is closely related to sepsis. The study aimed to determine the method of using animal models of sepsis diabetes through a combination of streptozotocin (STZ) and *Staphylococcus aureus* infection based on biological marker parameters.

**Materials and Methods:**

A total of 30 male Wistar rats of 2.5-3 months old weighing approximately 150-250 g body weight (BW) divided into six treatment groups with five replications per group were used in the study. Treatment A was negative control (healthy rats) and Treatment B was the positive control (with diabetes) where rats were given STZ dose at 45 mg/kg BW on day 8 intraperitoneally (IP). The blood glucose was measured on day 10, Treatment C was a positive control (bacteria), rats inoculated with *S. aureus* with a concentration of 10^8^ CFU/mL on day 8 given IP and observed sepsis conditions on day 10^th^. Treatment group (D, E, and F): Rats given STZ dose at 45 mg/kg BW on day 8^th^ by IP and measured blood glucose on day 10^th^, then inoculated with *S. aureus* with different concentrations of 10^5^ CFU/mL, 10^6^ CFU/mL, and 10^7^ CFU/mL on the 10^th^ day, respectively, and were later observed the condition of sepsis on day 12^th^. Data on diabetes bacteremia were quantitative used blood glucose levels, the bacterial count, and C-reactive protein (CRP) and were analyzed using the one-way analysis of variance test with a confidence level of 95%. Physical examination (temperature and respiration) is qualitative.

**Results:**

Physical examination showed that all treatments had a normal temperature, an increased pulse in Groups D, E, and F and a decrease in respiratory rate in the treatment of E and F, the bacteria found in the vital organs in all groups, and CRP levels were not significantly different at all.

**Conclusion:**

Animal model of diabetes sepsis can be observed through a combination of pancreas damage, and respiration, the bacteria in the vital organs.

## Introduction

Sepsis is a condition that damages the body, characterized by systemic activation of the inflammatory pathway and coagulation in response to microbial infections in ordinarily sterile parts of the body [1], and is often exacerbated by a number of conditions for metabolic disorders including type 1 and 2 diabetes mellitus (DM) [2]. Sepsis is characterized by loss of control of the inflammatory response, which can be triggered by various microorganisms and toxic secretions. The prevalence of sepsis in dogs showed 89 individuals (78%) had dysfunction in one or more organ systems, and 57 individual (50%) dogs showed multiple organ dysfunction organs. The mortality rate increases the number of dysfunctional organ systems [3]. In animal models of type 2 diabetes are known that the inflammation induced by more severe sepsis compared with no diabetes. Animal with sepsis diabetes also experiences an increasing number of bacterial infection and dysfunction in the expression of inflammatory cytokines and immune cells. The literature study on obese and diabetes models (given a high-fat diet) has a higher mortality rate when challenged with *Staphylococcus aureus* compared with no infection [1]. Increased mortality in animal models of diabetes occurs after 72 h associated with persistent bacteremia and reticuloendothelial microbial presence [4]. Diabetes that does not heal can cause complications such as neuropathy, vasculopathy, retinopathy, immune defects, and sepsis [5]. DM is considered a state of immunosuppression. Diabetes patients are very susceptible to endothelial dysfunction during sepsis. A recent study showed that E-selectin, leukocyte adhesion molecules dissolved, vascular cell adhesion molecule 1, intercellular adhesion molecule 1, vascular endothelial growth factor, and increased significantly in diabetes patients compared with patients without diabetes during the most severe sepsis stage. Some research suggests that patients with diabetes showed a clear activation of multiple pathways endothelium during sepsis, especially during severe sepsis. These molecules play a role in the inflammatory response during sepsis [4]. This suggests that patients with diabetes showed a clear activation of multiple pathways endothelium during sepsis, especially during severe sepsis [5]. Treatment of diabetes with a microbial infection is still unresolved, so the mortality rate is still high. This is estimated because the incidence of the disease is difficult to detect.

Animal models of diabetes and sepsis have developed their own to create a system that can be reproduced for studying the pathogenesis, preliminary testing of the potential therapeutic agent but animal models of diabetes bacteremia yet. Until now, the existing animal models are diabetes accompanied by foot infection or gangrene or diabetes foot [5], whereas there are no diabetes animal models with *S. aureus* infection.

The study aimed to determine the method of using animal models of sepsis diabetes through a combination of streptozotocin (STZ) and *Staphylococcus aureus* infection based on biological marker parameters.

## Materials and Methods

### Ethical approval

Maintenance and handling of Wistar rat animals in a laboratory were based on the letter of ethics no. 937-KEP-UB from Biosains, Brawijaya University.

### Research methods

This research is of true experimental laboratory post-control only design, which created an animal model of diabetes sepsis using a combination of STZ and *S. aureus*.

### Location and time of research

This research was conducted at the Pharmacology Laboratory of the Medical Faculty and Veterinary Medicine of Brawijaya University, Indonesia.

### Sample and population

The study sample used white rats stain Wistar used for diabetes sepsis STZ and *S. aureus* administered intraperitoneally (IP) in a completely randomized design.

### Experimental design

This study used male rat (body weight [BW] 150-250 g). The rat was previously adapted for 7 days. In this study consisted of six treatment groups, namely: Treatment A (negative control): Five normal/healthy rat, Treatment B (diabetes): Five rats were given STZ dose at 45 mg/kg BW on day 8^th^ IP and measured blood glucose on the 10^th^ day, Treatment C (bacteria): Five rats were inoculated with *S. aureus* with a concentration of 10^8^ CFU/mL on the 8^th^ day and observed sepsis conditions on the 10^th^ day, Treatment D: Five rats given STZ dose at 45 mg/kg BW on day 8^th^ IP and measured blood glucose on day 10, then inoculated with *S. aureus* with a concentration of 10^5^ CFU/mL on day 10^th^, and observed the condition of sepsis on the 12^th^ day, Treatment E: Five rats given STZ dose at 45 mg/kg BW on day 8^th^ IP and measured blood glucose on day 10, then inoculated with *S. aureus* with a concentration of 10^6^ CFU/mL on day 10, and observed the condition of sepsis on the 12^th^ day, and Treatment F: Five rats given STZ dose at 45 mg/kg BW on day 8 IP and measured blood glucose on day 10, then inoculated with *S. aureus* with a concentration of 10^7^ CFU/mL on day 10, and observed the condition of sepsis on the 12^th^ day.

### Induction of an animal model of type 1 diabetes rats

STZ (Cat. No. 41910012-4 [714 992], bioWORLD Dublin, Dublin, OH) 32.5 mg was dissolved in (50 mM, 0.1M, pH 4.5) buffer citrate to a final concentration of 32.5 mg/mL and preserved in a frozen condition before use. Animal treatment adapted in cages for 7 days, after the treatment of diabetes control and treatment (D, E, and F) was fasted overnight (6-8 h). The rats were further injected with a single dose of STZ via intraperitoneal route (45mg/kg BB) and blood glucose levels were measured 2 days after STZ injection. Rats with fasting blood glucose >270 mg/dl were considered diabetes positive. [6,7]. Blood sugar was measured using digital blood glucose level Glucostick (Gluco-Dr^®^) device.

### Bacterial culture

*S. aureus* bacteria were obtained from the Microbiology of FK Universitas Brawijaya. *S. aureus* bacterial identification using mannitol salt to be positive, catalase test positive, and Gram stain showed Gram-positive, cocci-shaped, and grape-clustered bacteria. The test bacteria have been resistant to several antibiotics, namely amoxicillin, vancomycin, cefoxitin, ceftriaxone, and penicillin.

Bacterial seeding was carried out by taking 10 colonies of *S. aureus* bacteria then cultured in Nutrient Broth media (Merck Millipore, Boston, USA) at 37°C for 24 h, then measuring optical density (OD) using a spectrophotometer. The results obtained with similar bacteria concentration of 0.1 OD 10^8^ CFU/mL and then made appropriate dilution for treatment [8].

### Preparation of animal model of sepsis

Wistar strain male rats were inoculated with 10^8^ CFU/mL of *S. aureus* through asepsis IP, and the bacterial dose was 2 mL per tail. Clinical signs, weight, and survival rates were monitored daily for 3 days after infection [8].

### Necropsy of an animal model

Necropsy begins with the administration of anesthesia using ketamine at a dose of 2 mg/kg bw (body weight) via intramuscular route per rat. After disinfection with 70% alcohol, surgical procedure was done to extract blood, liver, heart and kidney. The numbers of *S. aureus* bacteria in the collected organs were then examined.

### CRP test

Rat blood sample was taken after 3 days of infection and stored inside a 3 ml vacutainer. The blood sample was then frozen for 1 h at room temperature and centrifuged at 4000xg, 4 °C, 15 min to obtain serum. The serum was further analyzed for CRP test using a commercial kit (Life Diagnostics, West Chester, AS) [8].

### Histopathological examination

Sample from pancreas was collected on the 12^th^ day, fixed in formaldehyde at 10% with phosphate buffer (pH-7.4) and then post-fixed for 24 h, dehydrated, and embedded in paraffin. Sections, with 3-4 mm of thickness, were cut with a microtome [9].

### Statistical analysis

The diabetes sepsis data that were analyzed include clinical symptoms (BW, temperature, pulse, respiration), CRP examination and the number of bacteria present in vital organs (kidneys, liver and heart). The clinical signs were analyzed descriptively. Quantitative of blood glucose and CRP levels were then analyzed using the one-way analysis of variance test with a confidence level of 95% to determine the difference in the effect of treatment on making animal models of sepsis diabetes.

## Results and Discussion

### Animal model of type 1 diabetes rats

**Figure-1:**
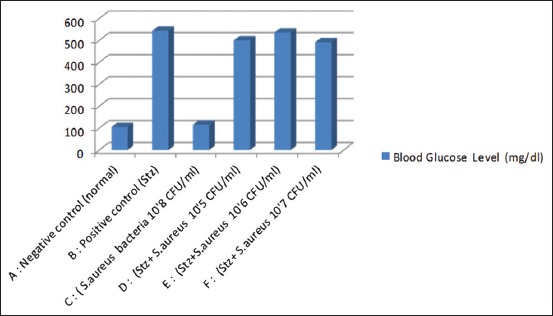
Blood glucose level.

The results showed that in the treatment of diabetes and diabetes sepsis showed blood glucose >270 mg/dL, whereas in normal treatment and sepsis that was not suspected STZ showed normal blood sugar levels (<120 mg/dl) (Figure-1). Blood sugar levels increase due to STZ induction. STZ is a β-cytotoxic drug, an antimicrobial agent and has also been used as an alkylating agent for acupuncture. STZ can cause pancreatic β-cell necrosis. The incidence of diabetes depends on animal species; the dose and route of administration from STZ are severe diabetes (blood glucose to 200/300 mg/dL) and mild diabetes (120-200/300 mg/dL). The pancreas can regenerate through the proliferation and neogenesis. Remodeling of the pancreas caused by increased replication and cell apoptosis on day 13-day 17. Under physiological conditions, the pancreas maintains glucose homeostasis [10,11]. Weight loss was only shown in STZ-induced rat while healthy rat and bacteria-induced rat did not show weight loss. Weight loss was significantly higher in the D and E treatment groups than in Groups B and F (unpublished data). This is in accordance with the research [8], which showed that rats induced by *S. aureus* bacteria concentrations of 4.5×10^4^-4.5×10^9^ CFU/mL showed no weight loss. According to the research conducted by Reis *et al*. [9], which showed that male Wistar rats induced by STZ at a dose of 65 mg/BW intravenous showed weight loss. The absence of insulin that serves to regulate the metabolism of sugars through the breakdown of sugars into simple molecules which are then distributed to the cells causes very high levels of glucose in the blood called as hyperglycemia. The body cannot use sugar as an energy source and stores extra glucose as fat, resulting in weight loss.

**Figure-2:**
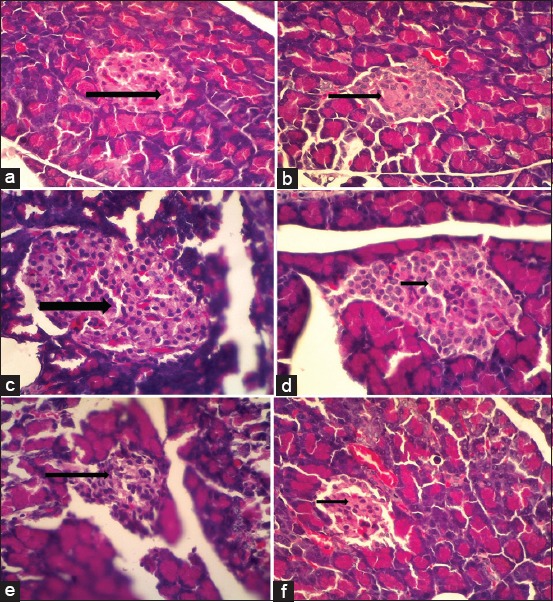
Images with magnifications 400×: Negative control (a) showing pancreatic beta-cell nucleus appears clear and spread evenly (arrow). Positive control diabetes (b) showed that the pancreatic beta-cell nucleus seemed to shrink even invisible and only seen cytoplasm (arrow). Positive control bacteria with a concentration of 10^8^ CFU/mL of *Staphylococcus aureus* (c) showing pancreatic beta-cell nucleus appear clear and spread evenly (arrow). Treatment diabetes and bacteria *S. aureus* with a concentration of 10^5^ CFU/mL (d) showed that the pancreatic beta-cell nucleus seemed to shrink even invisible and only seen cytoplasm (arrow). Treatment diabetes and bacteria *S. aureus* with a concentration of 10^6^ CFU/mL (e) showed that the pancreatic beta-cell seemed to shrink (arrow). Treatment diabetes and bacteria *S. aureus* with a concentration of 10^7^ CFU/mL (f) showed that the pancreatic beta-cell seemed to shrink (arrow).

STZ causes an increase in free radicals that serve to destroy the pancreatic β-cells. This result is quite interesting to observe that diabetes rats characterized by pancreatic β damage and turned out to be clinical symptoms that appeared normal. The results showed that in the diabetes control group (B) and all diabetes and bacterial treatments (D, E, and F) showed pancreatic beta-cell nuclei shrinking and even disappearing, only cytoplasm was seen so that the Langerhans island cell density was lower or less [9] than in the group healthy (A) and a positive control bacteria (C) showing pancreatic beta-cell nucleus appear clear and evenly so that a higher density of pancreatic cells (Figure-2).

STZ works by inhibiting the enzyme activity of free radicals, thereby increasing the formation of superoxide radicals, and nitric oxide turn produces reactive oxygen species (ROS) or oxidative stress which can cause oxidative damage to cellular components (lipids, DNA, and proteins) and trigger the activation of signaling pathways and disrupt standard repair mechanism. STZ enters the tissue through glucose transport, GLUT2 in the plasma membrane, and then, it will go to the pancreas and affect other organs such as the liver and kidneys. When the pancreatic β-cell is destroyed, insulin secretion decreases so that blood glucose increases and is not controlled in the blood. ROS can increase oxidative stress through increased production of p21 and reduced insulin messenger RNA cytosolic adenosine triphosphate and calcium flux in the cytosol and mitochondria [12].

### The existence of bacteria in vital organs

In this study, animal model was used to induce a combination of diabetes and sepsis. On bacterial examination, bacterial infections are found in vital organs in all treatments because bacteria would enter the blood vessels and would spread through lymphatic vessels to organs and cause multiple organ damage to death. Bacterial infections that enter the body cause hemodynamic changes that interfere with microcircular and cellular disorders resulting in the development of various organ dysfunctions and death. The bacteria were induced by IP in rats showed an increased pulse in all treatments (above 450 beats/min), respiration decreased only in the treatment Groups E and F (below 130 beats/min) while the temperature in normal conditions for all treatments (36.07-37.32°C). This is supported by research conducted by Popov and Paplov [13], which indicates that there are differences in the animal model of sepsis between positive and Gram-negative bacteria. Sepsis animal model was caused by the administration of gram-positive bacteria (intravenously), low hemodynamic appearance, and changes in lung disorder were observed. Whereas gram-negative bacteria causes hemodynamic shock and acute respiratory disorder.

*S. aureus* bacteria can replicate in the blood and colonize multiple organs and cause fatal sepsis [14]. Bacteria in the organs of diabetes rats are controlled by genes that encode toxins and protease enzymes that cause tissue damage [15]. Furthermore, there are genes related to virulence factors such as adhesion molecules, capsule polysaccharides, siderophore, and metabolic and transport systems of amino acids and carbohydrates that support the severity of endocarditis. Diabetes rats can interfere immunity which can accelerate the infection. According to Popov and Paplov [13], the development of sepsis animal models using rodents depends on the type of bacteria, route of administration, bacterial dose, and frequency of administration. The higher dose of the bacteria with a direct route into the blood vessel then will be more severe clinical symptoms.

Diabetes rat would be at high-risk exposure to pathogens and disease will be more severe. According to Mai *et al*. [16] states that high-fat feed-induced rat is at high risk of increasing the number of bacteria, decreasing the T-cell immune system to eliminate bacteria, and increasing pro-inflammatory and anti-inflammatory cytokines compared to normal mice. The study showed the death of rats in positive diabetes control and *S. aureus* bacteria concentration of 10^8^ CFU/mL on day 2 after bacterial induction. The results of this study are different from those conducted by Wu *et al*. [8] which showed rat died after being induced with *S. aureus* with successive concentrations ranging from 4.5×10^7^ to 4.5×10^9^ CFU/mL intravenously which were observed 7 days post-infection. Death in diabetic rats is due to the impaired immune system through decreased production and function of inflammatory cytokines, loss of phagocytic function, and body antioxidant production [17]. The high concentration of *S. aureus* induced in rat resulted in increased inflammation. Bacteria that enter IP will activate macrophages to do phagocytosis so that there will be an increase in the production of free radicals and inflammatory cytokines. However, death was not found in all diabetes treatments and *S. aureus* bacteria from concentrations of 10^5^-10^7^ CFU/mL. The high sugar levels and bacterial induction did not affect the clinical symptoms of rat even though bacteria were found in vital organs. When viewed from the results of the study, diabetes control rat had an average blood sugar level higher at 544 mg/dl compared to diabetes and bacterial groups.

### CRP test

CRP can be found in vertebrates (humans, mice, and rats) and invertebrate animals [18]. CRP is an acute inflammation produced in the liver as a result of responses to phagocytic cells that are affected by proinflammatory cytokines, namely interleukin (IL)-1, IL-6, and tumor necrosis factor-α. CRP will appear after 6-8 h after the initial infection and peak at 36-50 h thereafter. CRP is part of the ligand-binding plasma calcium-dependent family. The mechanism of action of CRP is that it binds to phosphocholine residues and then causes membrane damage and cell apoptosis. CRP will activate the classic path complement, C1q protein will then activate C3 and end with membrane damage [19].

**Figure-3:**
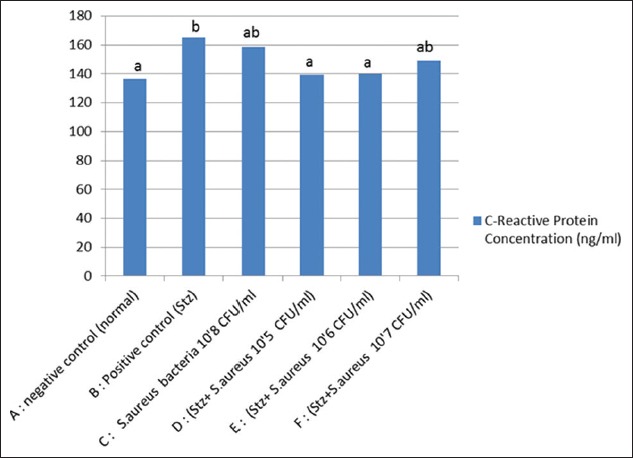
Level of C-reactive protein. Superscript letters are significantly different from one another based on analysis of variance with honestly significant difference tests (p<0.05).

The results showed that rat in the group of D, E, and F (diabetes rats with a concentration of *S. aureus* bacteria consecutively 10^5^ CFU/mL, 10^6^ CFU/mL, and 10^7^ CFU/mL) was significantly different from control diabetes but not significantly different from control *S. aureus* bacteria concentration of 10^8^ CFU/mL (Figure-3) and healthy control. This shows that CRP levels increase only in diabetes conditions, whereas in diabetes with diverse high concentrations of bacteria does not show an increase in CRP levels. The results are consistent with the research conducted by Dimitrov *et al*. [20], which showed that increased CRP could be detected in animals that have decreased in inflammation and an increase in high carbohydrate levels. CRP test demonstrates that the presence of acute inflammation such as in humans is less prominent in rats as a result of induction of bacteria does not activate the complement pathway [21]. This study contradicts the results of human studies, which showed that serum CRP levels were high in conditions of bacterial sepsis compared to healthy humans [22]. CRP in the rat is not specific to indicate an acute inflammatory reaction. Levels of CRP concentrations in rats would increase the basal metabolic condition that is approximately 300-500 mg/l, 100 times higher than in humans [18]. The biological effect indicates that the average of CRP level in healthy rat lower than that of all the treatment of diabetes and bacteria and also bacterial induction courses. This shows that the induction of diabetes and bacteria can trigger inflammation. At the time of entry of the antigen in the body, there will cause inflammatory cells out of the blood vessels leading to the injured area or damaged tissue, causing the release of inflammatory mediators to clear pathogens and wound healing agent [23].

CRP in the rat cannot activate complement which mediates inflammation except ligand-specific CRP mediated by C-polysaccharide from bacterium *Streptococcus pneumoniae* [18], but it can induce inflammation, pro-oxidants, and pro-coagulation through a pathway to increase macrophage activation [20]. Biomarkers of diabetes sepsis in Wistar rats can be seen from the weight loss, increased blood sugar, pancreatic cell damage, increased pulse, and decreased respiration, and the bacteria found in the vital organs.

## Conclusion

Biomarkers of animal models of sepsis diabetes using Wistar rats through a combination of weight loss increased blood sugar levels and pancreatic cell damage, increased pulse and decreased respiration, and found bacteria in vital organs in all treatments.

## Authors’ Contributions

DQ was responsible for controlling the course of studies, culturing of bacteria, and also analyzing data. DEA did CRP test; MAGB and AA did the analysis. All authors read and approved the final manuscript.
